# Supplementary material for: Identification and Expression Analysis of the WOX Transcription Factor Family in Foxtail Millet (Setaria italica L.)
Source: Genes (Basel). 2024 Apr 10;15(4):476. doi: 10.3390/genes15040476 (PMC11050393; doi:10.3390/genes15040476)
Supplement: Supplementary file 1 [file genes-15-00476-s001.zip › Figure S1. SiWOX protein amino acid multiple sequence alignment results...pdf]

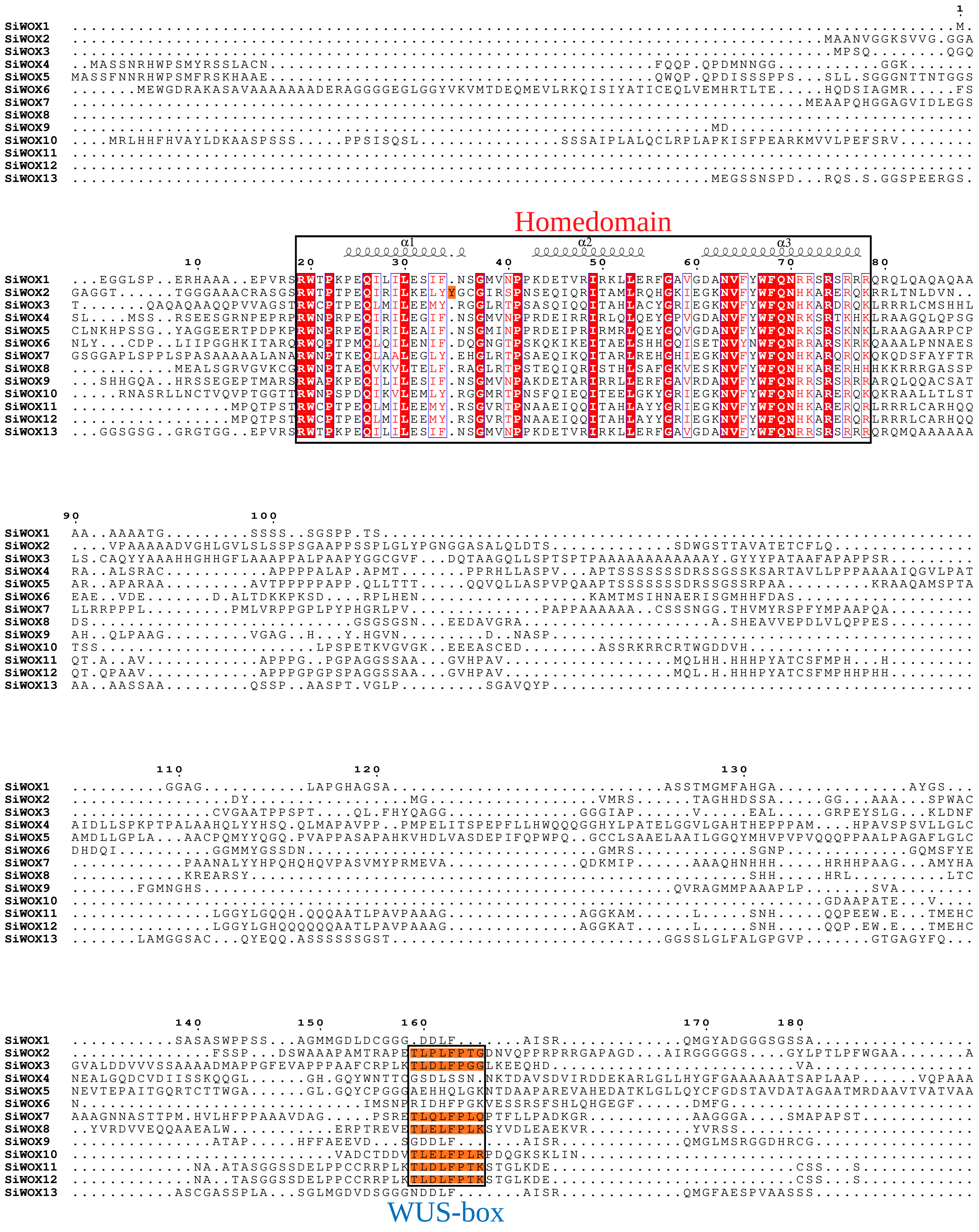
Homedomain
α1
α2
α3
WUS-box

EAR motif

```
                                            190       200                                         210            220
SiWOX1   ...................AAAVSQQQQHQQLYYSCQP.............................G......SMTVFINGVATEVPR..GPIDL
SiWOX2   TAATATTTTTS......VTIQQQ..HHQLLQLQDQY..SFYTTNSQPPSSHD.............ASSAATASLEL..SLSSWCSPYP..AGTM..
SiWOX3   ................................................................................................
SiWOX4   DASTAML..LP..SSAPSNVAAATSSVLTDQLQGLLDPGLIGGTPLPPPTATVVVVARDA..................VTCAVTAQFSVPAMRLDV
SiWOX5   TATTARLTGLPASSAAPNGVVA...NYDLMQLQGLAADGALGAGATTTSTGAPVAVATAAVAGEQQEGGGVAALCITDSVTGRSVAHSVAAARLDV
SiWOX6   ................................................................................................
SiWOX7   ..ASASFSGES......ESLEESPDSYSEALAVPFY..DFFGLQS.......................GGR.........................
SiWOX8   ..ASEQCR..........................EF..SFFDVAA.......................GRDPPLEL..RLCSFGP...........
SiWOX9   ...................YTASD.....ASQMTYQPT.............................G....MTSIQVFINGAVYEVPGAGAPLDL
SiWOX10  ................................................................................................
SiWOX11  ..KSSSCS.............TSTN.......................................................................
SiWOX12  ..KSSSCS.............TSTN.......................................................................
SiWOX13  ...................VA..PSTAGHQQQYYSCQS.............................P....AATITVFINGVPMEVPR..GPIDL
```

```
                   230                240       250       260
SiWOX1   RSMFGQDVMLVHSTGGL......LPVNEYGVLMQSLQMGESYFLVTRG.
SiWOX2   .................................................
SiWOX3   .................................................
SiWOX4   KLAFGDAAVLVRHTGEP......VLVDESGVTVEPLQQDTLYYLLMVTH
SiWOX5   RAQFGEAAVLFRCAGERGLDLEHVPVDASGCTVQPLQHGAFYYVLV...
SiWOX6   .................................................
SiWOX7   .................................................
SiWOX8   .................................................
SiWOX9   AGTFGHDAMLVHSSGEI......LPVNEHGVLMKSLQMGECYYLVSRST
SiWOX10  .................................................
SiWOX11  .................................................
SiWOX12  .................................................
SiWOX13  RAMFGQDVMLVHSTGAL......LPVNDYGILTQSLQMGESYFLVARPT
```
