# Supplementary figures and images for: Identification and Expression Analysis of the WOX Transcription Factor Family in Foxtail Millet (Setaria italica L.)
Source: Genes (Basel). 2024 Apr 10;15(4):476. doi: 10.3390/genes15040476 (PMC11050393; doi:10.3390/genes15040476)

motif 1

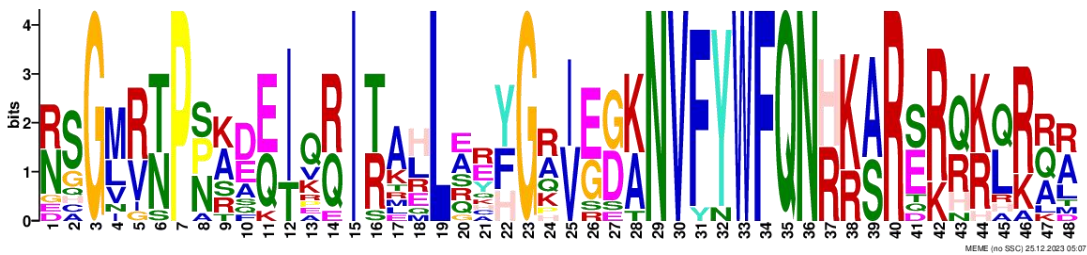

motif 2

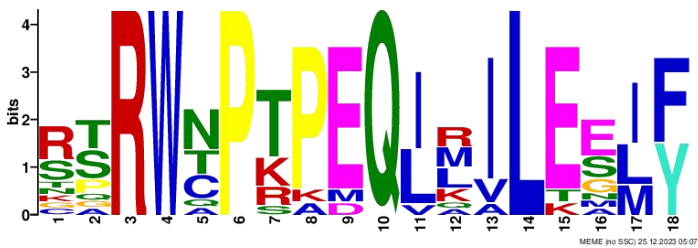

motif 3

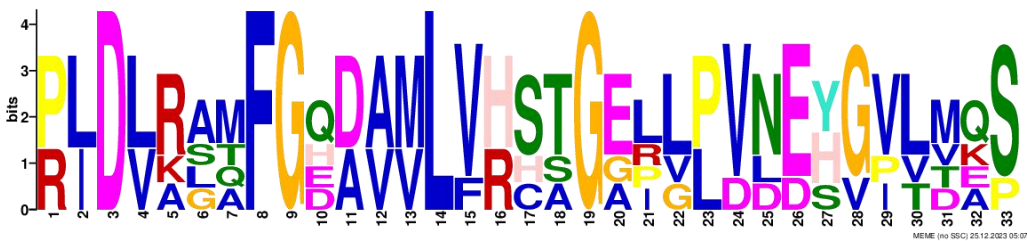

motif 4

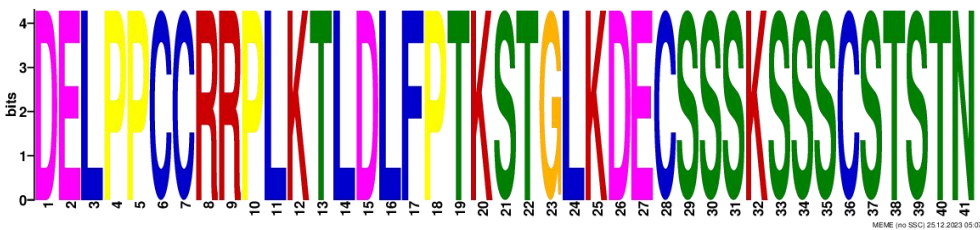

motif 5

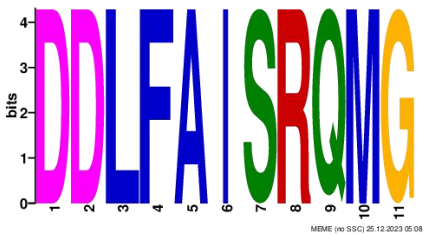

motif 6

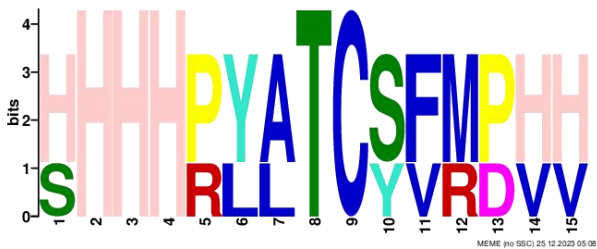

motif 7

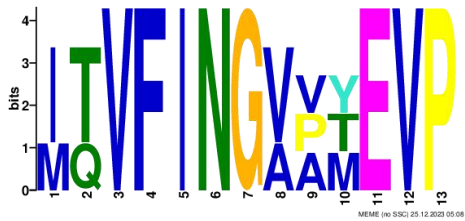

motif 8

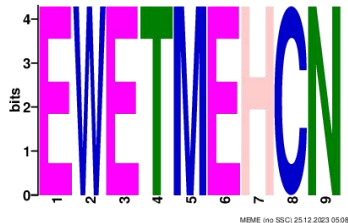

motif 9

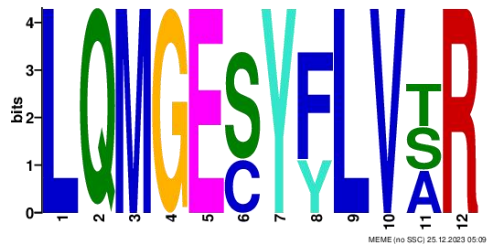

motif 10

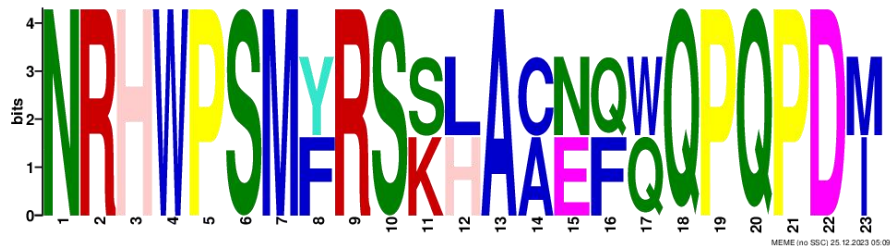

motif 11

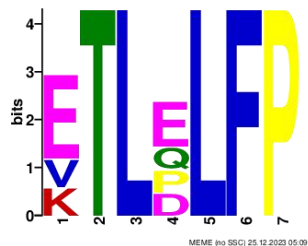

motif 12

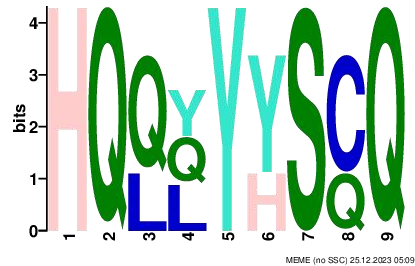

Supplement: Supplementary file 1 [file genes-15-00476-s001.zip › Figure S2. Weblogo plots of the 12 conserved motifs of SiWOX peptids..pdf]
